# The natural growth history of persistent pulmonary subsolid nodules: Radiology, genetics, and clinical management

**DOI:** 10.3389/fonc.2022.1011712

**Published:** 2022-12-08

**Authors:** Zhedong Zhang, Lixin Zhou, Fan Yang, Xiao Li

**Affiliations:** Department of Thoracic Surgery, Peking University People’s Hospital, Beijing, China

**Keywords:** subsolid nodules, growth history, early stage lung cancer, radiomics, multidisciplinary team

## Abstract

The high detection rate of pulmonary subsolid nodules (SSN) is an increasingly crucial clinical issue due to the increased number of screening tests and the growing popularity of low-dose computed tomography (LDCT). The persistence of SSN strongly suggests the possibility of malignancy. Guidelines have been published over the past few years and guide the optimal management of SSNs, but many remain controversial and confusing for clinicians. Therefore, in-depth research on the natural growth history of persistent pulmonary SSN can help provide evidence-based medical recommendations for nodule management. In this review, we briefly describe the differential diagnosis, growth patterns and rates, genetic characteristics, and factors that influence the growth of persistent SSN. With the advancement of radiomics and artificial intelligence (AI) technology, individualized evaluation of SSN becomes possible. These technologies together with liquid biopsy, will promote the transformation of current diagnosis and follow-up strategies and provide significant progress in the precise management of subsolid nodules in the early stage of lung cancer.

## 1 Introduction

With the widespread use of LDCT in lung cancer screening, lung cancer-related mortality has been significantly reduced (1, 2), and the detection rate of pulmonary nodules have increased dramatically, especially for SSN. SSN can be divided into pure ground-glass nodule (pGGN) and partially solid nodule (PSN) according to whether it contains solid components (3, 4). In Asia, LDCT is often used for screening purposes, even in non-smoking populations, where SSN is usually found (5, 6). However, the reported prevalence of SSN is lower in other parts of the world. At the same time, most data come from randomized controlled lung cancer screening studies in which most participants were smokers (7, 8). Recommendations vary across guidelines due to limited data on SSN management (9–11), and nodules of different sizes and densities have different follow-up frequencies and clinical treatment strategies according to the unique natural history of persistent SSN. The appropriate diagnostic and management strategy for those lesions puts physicians in a dilemma. This article will review, and analyze the evidence to date on the natural history of SSN and provide evidence-based recommendations for the management of SSN.

## 2 Different considerations

Some pulmonary nodules with SSN as the primary manifestation are transient, and the pathological type may be inflammation, hemorrhage or interstitial lung disease (12, 13). Meanwhile, the persistence of a nodule has significant implications upon differential diagnosis, including benign or lung adenocarcinoma (LUAD) and its precancerous lesions, including invasive adenocarcinoma (IAC, mucinous and non-mucinous), minimallyinvasive adenocarcinoma (MIA), adenocarcinoma *in situ* (AIS) or atypical adenomatous hyperplasia (AAH), and often have features distinct from those of solid cancers (14) (**Table 1**).

**Table 1:** Differential diagnosis of SSN.

	Transient	Persistent
Benign	Infection (aspergillosis, candidiasis)	Focal interstitial fibrosis
	Inflammation	Organizing pneumonia
	Drug reaction	Endometriosis
		Focal interstitial fibrosis
Malignant		Mucosa-associated lymphoid tissue (MALT)
		Atypical adenomatous hyperplasia
		Adenocarcinoma in situ
		Minimally invasive adenocarcinoma
		Invasive adenocarcinoma
		Mucinous adenocarcinoma
		Metastatic lesions

Cho et al. (15) performed surgery on 39 patients with persistent and stable pGGN at follow-up. Postoperative pathology revealed three benign lesions (each with fibrosis, hemorrhage, and metaplasia), 1 case of IAC, 1 case of MIA, 21 cases of AIS and 13 cases of AAH, from which more than 90% of persistent pGGNs are lung cancer-related lesions. Ye et al. (16) reported that 92.6% of the persistent SSN patients were eventually confirmed to be malignant. Kim et al. (13) reviewed 293 persistent metastable nodules surgically removed at a single center and found that 77.5% were pathologically diagnosed with lung cancer. To date, 14 studies have reported pathological outcomes of SSNs after long follow-ups, with a cumulative total of 329 SSNs, summarized in a meta-analysis (17). Of these, only 4/329 (1.2%) were benign, including 3 interstitial fibrosis and 1 pulmonary capillary hemangioma lesion. A total of 325/329 (98.8%) SSNs were pathologically proven to be lung cancer or precursor gland lesions. 307/329 (93.3%) SSNs were lung adenocarcinoma or precancerous lesions, 2 were pleomorphic carcinoma or squamous cell carcinoma, 5 were broncho-alveolar carcinoma, and the other 11 SSNs were unclassified. It is not difficult from the above data that persistent GGO often predicts malignant lesions.

## 3 The nature growth history

**Figure 1:**
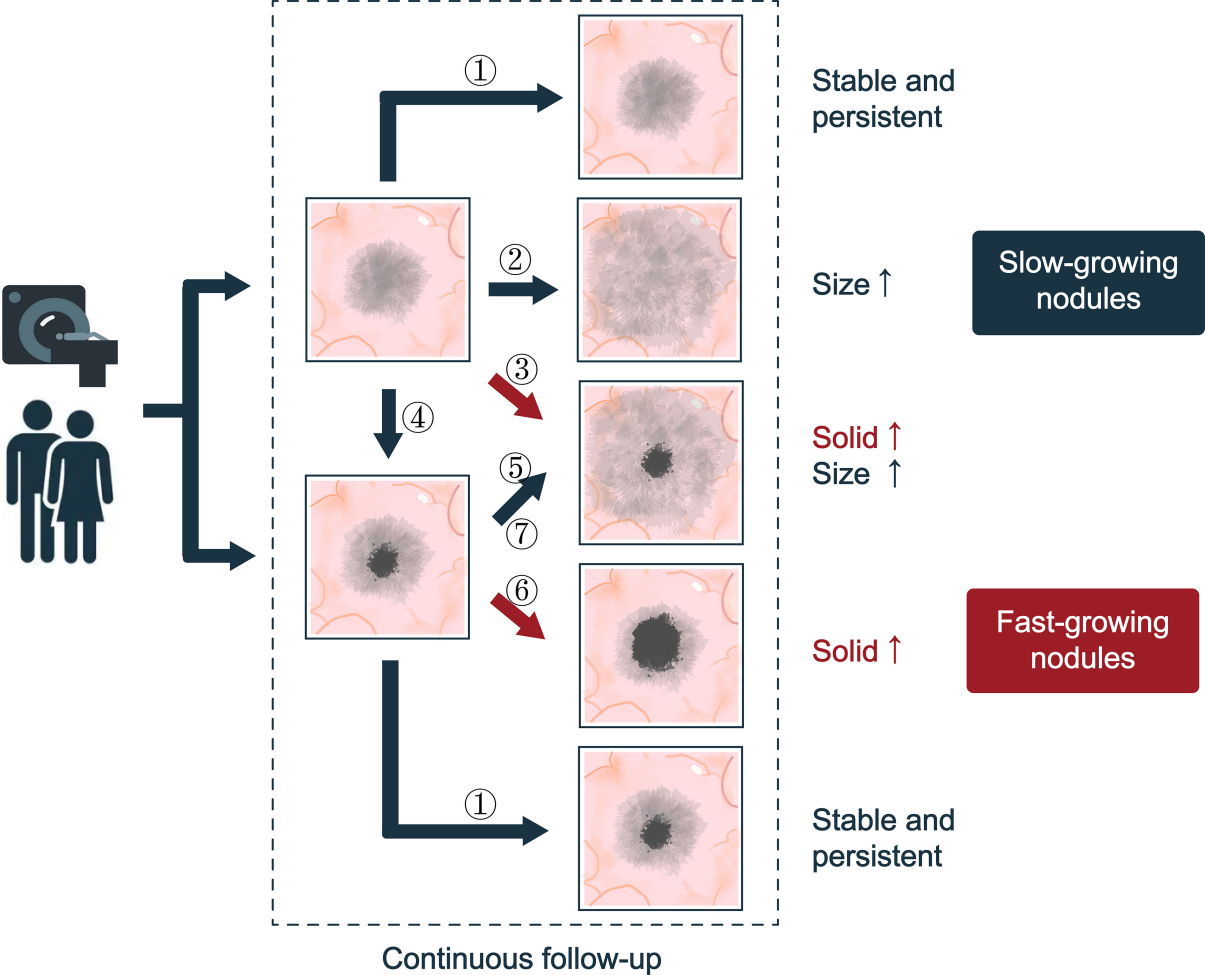
The natural history of SSNs. Seven types of progression are suggested: ① No changes; ② Increased ground glass composition in pGGN, and there is no solid component; ③ The diameter of the pGGN increases, and a solid component is produced; ④ the solid component appears with no changes in pGGN size; ⑤ Increased ground glass composition in mGGN and the solid component did not chang; ⑥ Increased solid composition in mGGN and the ground composition did not change; ⑦ The solid and ground glass component of mGGN are increasing. According to the growth rate of ground glass and solid components, SSNs can be divided into fast-growing nodules and slow-growing nodules.

Growth of the SSN is a strong predictor of malignancy in the lung and is usually defined as an increase in nodule diameter or volume on serial chest CT scans (18), which can be measured using electronic calipers or semi-automatic tools. And the nature growth history of SSN is summarized in **Figure 1**. However, due to the inherent limitations of measurement tools, determining actual nodule growth with a slight increase in nodule size can be challenging. It has been reported in the literature that the measurement error in SSN is 1.72 mm for manual measurement and 2.1 mm for machine-assisted semi-automatic measurement (19, 20). The Fleischner Society guidelines define the growth criteria for SSN as (1) an increase in the diameter of the nodule or solid component by more than 2 mm; (2) the appearance of a new solid component in the nodule (21). In contrast, the British Thoracic Society (BTS) nodule management guidelines stipulate a 25% increase in nodule volume to determine growth (22). However, its limitation is that when measuring the volume of pulmonary nodules in clinical practice, the nodules are often subjectively analyzed as spherical structures. The long and short diameters of the nodules were measured several times on the CT image, and the average value was taken as the diameter of the ball, and then the nodule volume was calculated. With radiomics, while increasing accuracy, it also increases the workload. At the same time, it has been reported that the error range of >5 mm GGN volume measurement is -27.3% to 29.5% (mean, 1.1%) (23).

### 3.1 The growth pattern—exponential growth

In solid malignancies, a doubling of cancer cells can translate into a doubling of tumor volume based on the constant cell division of cells at a steady rate. In LUAD, characterized by SSN, cancer cells grow predominantly by attaching to the alveolar wall (24). Previous studies assumed exponential SSN growth and used volume double time (VDT) and mass double time (MDT), the time it takes for a tumor to double in volume or mass, to assess SSN growth (25, 26). However, there was no study further to verify the reliability of VDT at that time. In 2020, Mellon et al. (27) reviewed 74 single-center SSN patients with pathologically confirmed LUAD with more than three preoperative chest CT imaging data. This study modeled the total volume of all SSNs and the solid component volume of the PSN and demonstrated that lung adenocarcinoma with SSN presentation on chest CT showed exponential growth in total volume and solid component. Through follow-up of growing SSNs, Qi et al. (28) found that the volume growth rate of SSNs with pathologically confirmed IAC remained almost unchanged in the first 20 months and then increased slightly; For SSNs with pathologically confirmed AAH/AIS/MIA, the volume growth rate remained virtually unchanged for the first 25 months, followed by a significant increase. This demonstrates that VDT is helpful for objectively evaluating GGO-predominant lesions’ growing tendency.

### 3.2 The growth rate—indolent growth

The exponential increase in SSN justifies using VDT and MDT to assess nodule growth. Overall, the VDT of SSN was more than 400 days, the median VDT for invasive adenocarcinoma was 631 days, and the median VDT for AIS/MIA was 802-811 days (26). Song et al. (23) found that pGGN, PSN fundamental component <5 mm, PSN fundamental component >5 mm, and their average VDT were 1 832.3 d, 1 228.5 d, and 759 d, respectively. The mean MDT was 1 556.1 d, 1 199.9 d and 627.7 d, respectively. The results showed that the PSN with fundamental composition >5 mm grew faster than the other two groups. Meanwhile, the study noted that the median VDT for AIS, MIA, and IAC was 1240.3 days, 1328.3 days, and 941.5 days. The median MDT for AIS, MIA and IAC were 1004.6 days, 848.2 days, and 782.5 days. The difference in median VDT and MDT among the three was not significant, and this study was the first to incorporate MDT into the nodule growth evaluation index. Qi et al. (28) included 95 SSNs and followed up for more than two years. Among them, 68 SSNs grew, with mean VDT: 1704.7 ± 1493.7 days, median MDT: 1294.1 days, mean growth time: 865.9 ± 570.9 days, and the meantime for pGGN to develop to PSN was 1145.5 ± 682.6 days. The limitation of this study that SSN was not further classified to describe VDT and MDT, respectively. Other studies have reported similar results: the average VDT range for pGGN and PSN was 769 to 880 days, and 277 to 457 days (29–32). SSNs grow indolently, and pGGNs generally have longer doubling times and slower growth rates than other types of nodules. In a meta-analysis, the duration of follow-up was not significantly associated with SSN growth or pGGN growth. After long-term stabilization, the frequency of SSN size increase was very small, so it could be speculated that the frequency of CT examination could be reduced for SSN follow-ups of more than 5 years (33).

## 4 Factors affecting the growth of SSN

Reviewing the relevant literature on the risk factors of SSN growth published in the past ten years, most are single-center retrospective studies, and the number of included nodules is more than 100 cases (**Table 2**). Patients with a history of lung cancer are at high risk for nodular growth. Regarding the morphological characteristics of nodules, current studies have confirmed that the size of the solid component in the nodule is an independent risk factor for nodule growth. Among the CT quantitative factors, the primary nodule diameter and CT value were the main ones.

**Table 2:** Summary of studies on the natural history of persistent Subsolid Nodules.

Author, Publication Year, and Reference No.	Country/Type of study	Patients/No. of nodules	Type of SSN/No. of nodules	Size of baseline SSNs (mm)	Follow-up period (month)	Growth definition and pattern	Time to progression (month)	Progression proportion (%)	Risk factors of SSNs progress	Note
Kim YW et al., 2021 (13)	Korea/Single-center, retrospective	4545/6725	pGGN 5116 mGGN 1609	pGGN 6.1 ± 2.1 mGGN 9.7 ± 5.2	Median, 35.1 (range, 13.7–58.5)	pGGN: De (n = 1317), I+IS+NS (n = 160); mGGN: De (n = 799), I+IS+NS (n = 130)	NA	pGGN 4.6% mGGN 9.2%	age, initial diameter, initial presentation as a part-solid nodule	SSNs detected at baseline and new-onset
Gao C et al., 2020 (34)	China/Single-center, retrospective	85/110	pGGN 83 mGGN 27	total 8.1 ± 3.8	≥24	NA	NA	pGGN 24.1% mGGN 59.2%	Lung-rad score, the initial diameter	SSNs followed for > 2 years
Qi LL et al., 2020 (35)	China/Single-center, retrospective	110/110	pGGN 110	8.7 ± 3.2	48.7 ± 23.8	pGGN: V (n = 52)	28.4 ± 22.5 (range, 3.8-95.5)	47.30%	lobulated sign, initial mean diameter, initial volume, initial mass	Persistent pGGNs follow-up of ≥ 2 years and those with a follow-up of < 2 years but that had grown
Lee JH et al., 2020 (36)	Korea/Single-center, retrospective	235/235	pGGN 211 mGGN 24	pGGN 8 ± 1 (range, 6–13) mGGN 9 ± 3 (range, 7–17)	Median, 112 (range, 84–208)	pGGN: I (n = 2), NS (n = 1); mGGN: IS (n = 2)	Median, 99 (range, 84-146)	pGGN 1.42% mGGN 8.3%	NA	GGNs with stability for 5 years and follow-up for at least 2 years, GGNs≥6 mm
Lee HW et al., 2019 (37)	Korea/Single-, center, retrospective	160/208	pGGN 162 mGGN 46	GGNs 4.7 (range, 1.7–10.0) solid component 2.1 (range,0.9–5.7)	Median, 136 (range, 120–179)	I+DS (n = 16); I (n = 11)	Median, 103 (range, 60–141)	pGGN 11.7% mGGN 17.4%	bubble lucency, history of cancer other than lung cancer, development of a new solid component	Stable for 5 years and follow-up for at least 5 years, 76% (159/208) of GGNs<6 mm, 70% of growing GGNs (19/27) <6 mm
Sawada S et al., 2017 (38)	Japan/Single-center, retrospective	226/226	pGGN 164 mGGN 35	Median, 10 (range, 3-30)	NA	NA	Median, 24 (range, 3-108)	total 17.3%	CTR	SSNs ≤ 30 mm
Tang EK et al., 2019 (39)	China/Single-center, retrospective	128/128	pGGN 93 mGGN 35	pGGN 7.14 ± 4.52, mGGN 17.52 ± 8.65	42.84 ± 35.16	pGGN: TG (n=37), SG (n = 17), SS (n = 4); mGGN: TG (n=37), SG (n = 17), SS (n = 4);	TG: Mean pGGN: 83.4, mGGN:47.5 SG: Mean pGGN: 113.1, mGGN:47.5 SS: Mean pGGN: 146.0, mGGN:86.4	TG: pGGN: 40.0%, mGGN:65.7% SG: pGGN: 18.2%, mGGN: 65.7% SS: Mean pGGN: 4.3%, mGGN:17.4%	older age, PSN nodule type, longer follow-up time	NA
Cho J et al., 2016 (40)	Korea/Single-centre, retrospective	218/453	pGGN 438 mGGN 15	Median, 5.0 (range, 2.0–31.1)	Median, 77.5 (38.1–117.1)	pGGN: I + NS (n = 1), NS + De (n = 1); mGGN: I + IS (n = 1); I (n = 12)	VDT 39.9(19.1-349.5)	total 3.3%	age 65 years or older, history of lung cancer, initial size 8 mm or larger, presence of a solid component, air bronchogram	Stable for 3 years and follow-up for at least 2 years
Kakinuma R et al., 2016 (26)	Japan/Multicenter, prospective	795/1229	pGGN 1046 hGGN 81 PSN 104	Median, 7.0 (IQR 5.5–9.0)	51.6 ± 30 (Median 42, IQR 28.8–72years)	NA	pGGN 45.6 ± 24 hGGN 25.2 ± 27.6	pGGN 16.6% hGGN 19.8%	initial diameter	SSNs ≤ 30 mm confirmed as persistent on follow-up
Scholten ET et al., 2015 (7)	Netherland/Multicenter, prospective	108/117	pGGN 69 mGGN 48	pGGN 11.1 ± 3.6 (range, 5.1–22.6) mGGN 12.7 ± 6.2 (range, 4.6–34.3)	Median, 95 (range 20–110)	pGGN: M (n = 26); mGGN: M (n = 35)	MDT<13.3 months 8.9%, MDT>13.3 months 51.4%	increase in mass≥30% pGGN 63.4%, mGGN 58.3%	NA	SSNs detected by NELSON
Eguchi T et al., 2014 (41)	Japan/Single-center, retrospective	124/124	pGGN 124	7.4 ± 2.8	Median, 57.0 (range, 24.1–113.6)	pGGN: NS (n = 40), I (n = 24)	Median, 38.0 (range 3.1–80.0)	51.61%	smoking history, initial tumor size, the mean CT attenuation value	SSNs followed for > 2 years
Song YS et al., 2014 (23)	Korea/Single-center, retrospective	97/97	pGGN 63 mGGN with solid parts ≤5mm 23 mGGN with solid parts >5mm 11	pGGN: Median, 8.3 (range, 5.5-22.2) mGGN with solid parts ≤5mm: Median, 11.1 (range, 6.5-19.3) mGGN with solid parts >5mm: Median, 18.8 (range, 14.5-23.8)	Median, 21.1 (range, 3.1–89.0)	pGGN: V (n = 12), M (n = 26); mGGN: V (n = 17), M (n = 20)	VDT:pGGN: Median, 61.1 (range, 41.0–151.0) mGGN with solid parts ≤5mm: Median, 40.9 (range, 31.2–153.9) mGGN with solid parts >5mm: Median, 25.3 (range, 12.5–31.4)	pGGN 27.0% mGGN with solid parts ≤5mm 39.1% mGGN with solid parts >5mm 81.8%	NA	NA
Kobayashi Y et al., 2014 (42)	Japan/Single-center, retrospective	67/120	pGGN 91 mGGN 29	Total: Median, 9 (range, 4–24)	Median, 50.4 (range, 6–144)	pGGN: D (n = 20); mGGN: D (n = 14)	within 36 months	pGGN 16.48% mGGN 65.52%	Smoking history, initial lesion diameter	The inclusion criteria for mGGN is that the solid component ≤ 50%
Kobayashi Y et al., 2013 (43)	Japan/Single-centre, retrospective	61/108	pGGN 82, mGGN 26	9.5 (range, 4–25)	Median, 50.4	SSN: I (n = 15); mGGN: I+ IS (n = 14)	NA	total 26.8%	NA	GGNs ≤ 30 mm confirmed as persistent on follow-up
Chang B et al., 2013 (29)	Korea/Single-center, retrospective	89/122	pGGN 122	Median, 5.5 (range, 3.0-20.0)	Median, 59 (range, 25-140)	pGGN: I (n = 12)	VDT: median, 25.6 (range, 11-101)	GGNs 9.8% People 13.5%	initial diameter, internal solid portion	pGGNs followed for > 2 years
Silva M et al., 2012 (8)	Italy/Multicenter, prospective	56/76	pGGN 48 mGGN 28	7.0 ± 2.0	50.26 ± 7.3	pGGN: DS (n = 0), D (n = 7), D+DS (n = 1); mGGN: DS (n = 3), D (n = 5), D+DS (n = 4);	NA	pGGN 16.7% mGGN 46.2%	NA	SSNs detected by MLID
Hiramatsu M et al., 2008 (44)	Japan/Single-center, retrospective	125/125	pGGN 95, mGGN 30	Total: Mean 8.3 (range, 3.0-17.0)	Mean, 34.9 (range, 5.9-109.0)	pGGN: D (n = 8), NS (n = 6); mGGN: D (n = 5), DS (n = 7)	Growth incidence at 3 and 5 years were estimated to be 18% and 30%, respectively	pGGN 14.74% mGGN 40.0%	the initial size, history of lung cancer.	SSNs were stable for 3 months

pGGN, pure ground-glass nodule; mGGN, mixed ground-glass nodule; hGGN, heterogeneous ground-glass nodule; PSN, part-solid nodule; SSN, subsolid nodule; IQR, interquartile range; VDT, volume double time; MDT, mass double time; NELSON, Nederlands Leuvens Longkanker Screenings Onderzoek; MILD, Multicentric Italian Lung Detection trial; I, increase in mean/longest diameter≥2mm; IS, increase in the solid portion of 2mm or more; NS, the emergence of a new solid component; 3D, increase in 3D diameter≥2mm; V, increase in volume by at least 20% or 25%; V/M, an increase of at least 30% in volume or mass; De, nodules that disappeared, or exhibited a decrease of ≥2 mm in the total size or solid portion; TG, true growth was defined as I+IS+NS; SG, substantial growth was defined as an obvious increase of ≧ 5 mm or more in SSNs, the solid portion in PSN increased by 5 mm or more from the baseline; SS, stage shift was defined as the specific stage shift of LUAD diagnosed to detect obvious different categories/stages shift according to the seventh lung cancer TNM staging system based on their clinical and pathologic information at the presentation of follow-up CT scan from the baseline.

However, it is worth noting that statistical analysis on the SSN growth pattern and rate suffers from two main problems. First, some patients had simultaneous multiple SSNs. When all SSNs were counted independently, patient characteristics such as gender, smoking status, or previous cancer history were overestimated due to the calculation of duplicate samples. Second, the definition of “no growth” as an ending time is still controversial, as some SSNs that do not grow for a certain period may start to grow later.

### 4.1 Clinical factors

During the follow-up of the SSN. Older age, patients with a history of malignancy and smoking are at high risk for nodular growth. Older age at screening detection of SSN was found to be significantly associated with a higher rate of lung cancer diagnosis (13, 39). Age over 65 years was an independent risk factor for SSN growth in the SSN with a stable presence beyond three years (40). Matsuguma et al. (45) found that the prior record of lung cancer was an independent risk factor for pGGN rather than PSN for the previous history of malignant tumors.

In pGGN, Tamura et al. (46) reviewed 63 cases to assess the relationship between clinical and imaging findings and pulmonary GGN progression, identifying risk factors that predict pGGN lesions. In the growth group, pGGN lesions were closely related to the high mean CT values and lung cancer history but not to smoking habits and GGN shape. Some studies have shown that a history of lung cancer is a predictor of the growth of the SSN with a persistent solid component ≤5mm (47). Furthermore, it was found that a history of malignancy other than lung cancer was a risk factor for predicting the growth of the SSN that had been stable for more than five years (37).

Smoking significantly increases the risk of many cancers and is highly associated with a poor prognosis. It is highly associated with a poor prognosis (48), and several studies have shown that smoking history is an independent risk factor for SSN growth (41, 42). Previous extensive screening studies recruited mainly Caucasian smokers, but for Asian populations, SSN was more prevalent in women and non-smokers (10). 4545 patients who underwent LDCT lung cancer screening at a single center, and a total of 6725 subsolid nodules were detected. Multivariate analysis showed that smoking history was not an independent risk factor for nodule growth. The study also pointed out that in Asian populations, especially those who have never smoked, subsolid nodules detected by screening require more careful and long-term follow-up (13). Studies suggest that smoking is not an independent risk factor for SSN growth in the Chinese population (49). Meanwhile, smoking history is not an independent risk factor for nodule growth in a long-term follow-up cohort of Japanese and Korean people with persistent SSN (26, 37). Therefore, for different ethnic groups, future multi-center studies with larger sample sizes are needed to explore the follow-up management of SSNs.

### 4.2 CT non-quantitative analysis factors

For persistent PSN, accurate assessment of its development and changes is inseparable from precise imaging examinations. Among the non-quantitative morphological features of CT, the current study confirmed that pGGNs were irregular in shape, with lobulated vacuolar and vascular signs being an independent risk factor.

**Figure 2:**
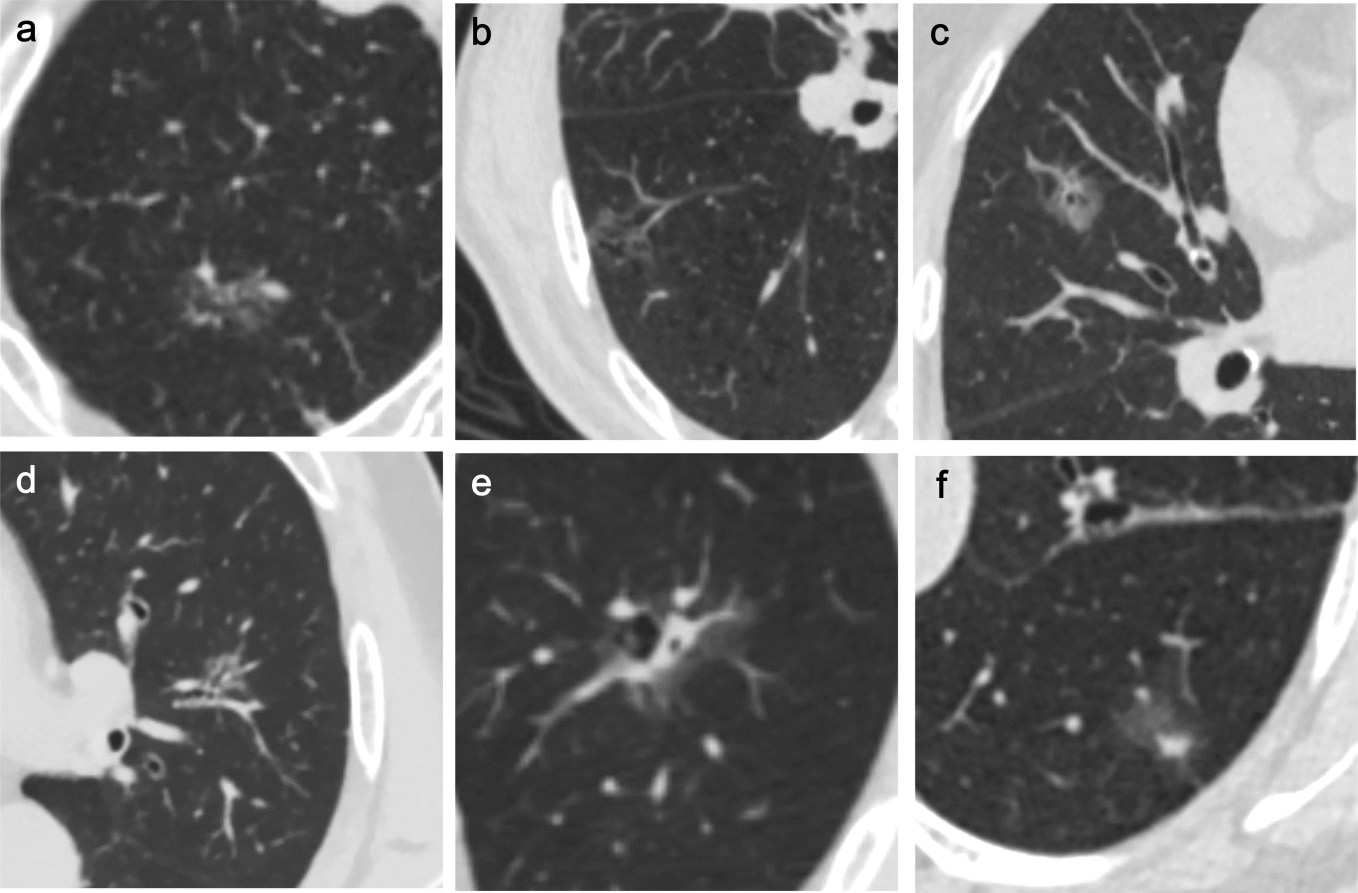
Independent risk factors for SSN growth in CT non-quantitative factor analysis. The six imaging features mentioned in the article are as follows: **(A)** lobulated sign, **(B)** vacuole sign, **(C)** vascular sign, **(D)** air bronchogram, **(E)** vacuole sign in PSN, **(F)** size of the solid component in the nodule.

Current studies have confirmed that pGGNs are irregular in shape, with lobulated (35), vacuolar and vascular signs in PSN, vacuolar signs (37), air bronchus signs (40), and the size of solid components in the nodule are independent risk factors (13, 38, 40) (**Figure 2**). Therefore, long-term monitoring and follow-up should be performed when persistent PSN has the above characteristics.

### 4.3 CT quantitative analysis factors

#### 4.3.1 Two-dimensional measurement index

Several previous studies have confirmed that the initially diagnosed diameter of PSN is one of the leading indicators for predicting the growth of nodules (29, 41, 45). In pGGNs, diameter ≥ 10 mm was a significant risk factor for increased GGN size in 175 GGNs of 114 patients (31). In addition, several studies have shown that the initial diameter of pGGN ≥ 10 mm is significantly associated with nodule growth and is highly correlated on the degree of malignancy of the nodule (50, 51). For PSNs with a solid component less than 5 mm, the growth rate of the initial diameter ≥ 8 mm is significantly higher than that of < 8 cm (47). For PSN with a solid component more significant than 5 mm, due to the presence of surgical indications, most patients choose surgical resection after discovery, and few select follow-ups. However, some studies have found that the initial diameter of SSN has no significant correlation with whether the nodule grows or not and the nodule growth rate (32, 46, 52, 53). The reasons can be classified into two points: (1) Diameter as a two-dimensional parameter has certain limitations. For example, most nodules are asymmetrically shaped, and their diameters cannot accurately represent the entire SSN (54). (2) Lead time bias may be due to the small sample size and different lengths of follow-up (55, 56).

#### 4.3.2 Three-dimensional measurement index

**Figure 3:**
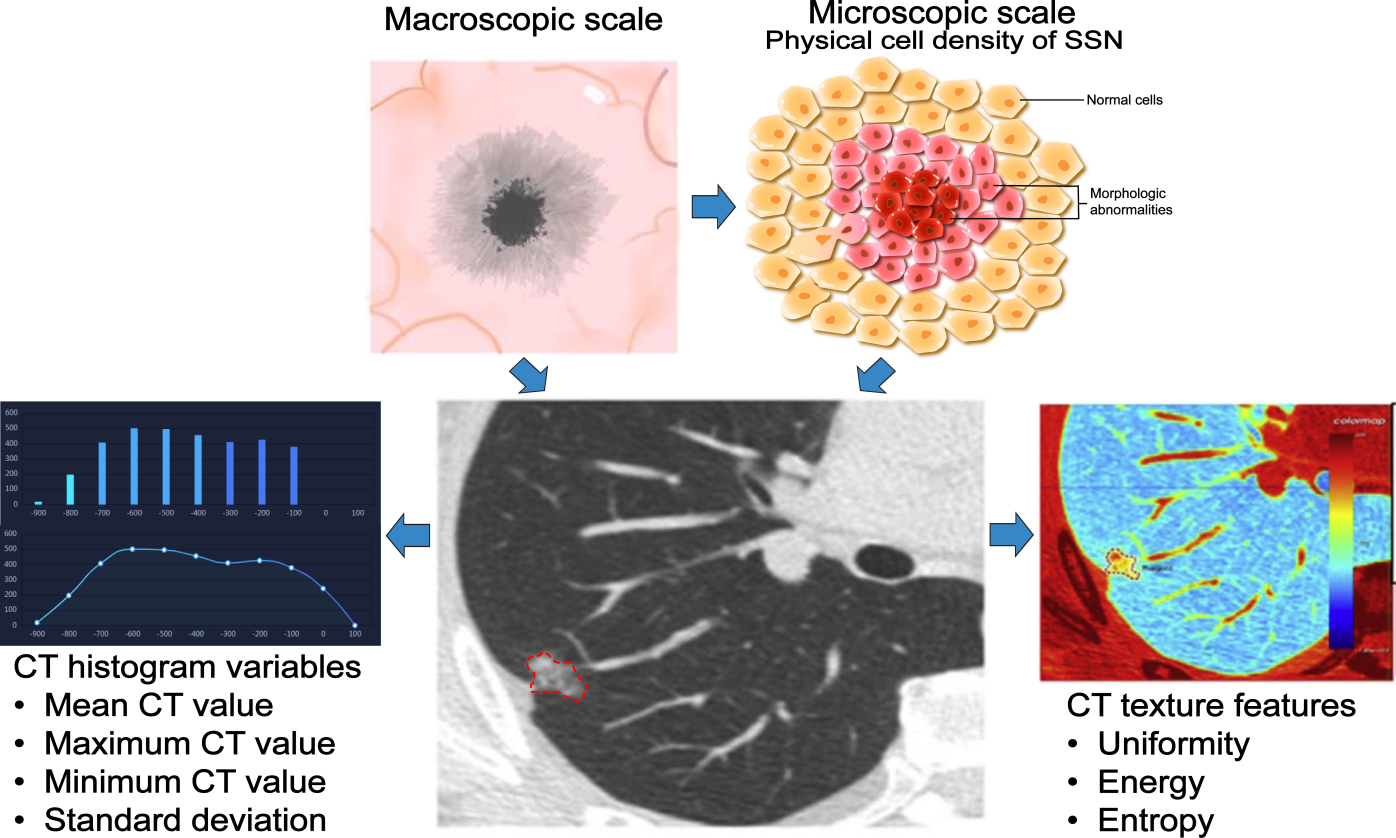
Application of three-dimensional measurement index in SSN. The performance of the SSN is determined by the physical cell density and atypia, and the CT value can reflect the physical cell density of the SSN in a macroscopic form. With CT manifestations, SSNs growth can be predicted based on CT histogram (mean CT value, maximum CT value, minimum CT value, standard deviation of CT value) and texture features (entropy, uniformity, and energy), resulting in better management of nodules.

The CT value of PSN is related to the physical cell density (57). Most AIS/MIA is mainly manifested by ground-glass opacity (GGO) (58), and IAC can be embodied as PSN or solid nodule (SN) (59). According to the growth pattern, SSNs with larger CT values (cut-off value about -670Hu) are more likely to overgrow or be pathologically malignant (41, 46, 52). However, if the growth of the GGO component in part-solid GGN is greater than the growth of the solid element, it may decrease the mean CT value, resulting in a decrease in the mean CT value of the PSN during follow-up (53). Therefore, using the mean CT value to predict pGGN growth may be more reliable than predicting PSN growth. The average CT value only represents the overall density of the nodule, and the maximum CT value, the standard deviation of the CT value, and the CT value histogram can better reflect the information on the heterogeneity of the SSN (60). SSNs with high heterogeneity are often malignant and aggressive (61, 62). Sun et al. (63) extracted CT texture features (CT mean, entropy, uniformity, and energy) of 89 SSNs followed for more than two years and found that the uniformity of the growing group in pGGN was significantly lower than that of the non-growing group. Meanwhile, this study found a positive correlation between uniformity and VDT, which indicated that pGGN with low uniformity had higher heterogeneity, faster growth rate, and malignant tendency (**Figure 3**). Therefore, mean CT value, maximum CT value, standard deviation, histogram and texture features of CT can be used to predict SSN growth. 

## 5 Characteristics of persistent and stable SSN

In clinical practice, long-term persistent and steady SSN is often encountered, and the nature of its nodules and follow-up strategies have always been a problem for clinicians and radiologists. In 2013, Kobayashi et al. (43) included 108 SSNs ≤3 cm and GGO ratio ≥50% for the study, and 29 nodules began to grow within three years after their first discovery, pointing out that all SSNs should be observed for at least three years. Then Cho et al. (40) analyzed the SSN that was stable within three years and continued to follow up to 5 years and found that based on population analysis, the probability of subsequent SSN growth was 6.7%, and based on nodule analysis, the likelihood of SSN growth was 3.3%, suggesting that longer follow-up is required. Time to confirm the continued stable growth of SSN, especially for people aged ≥ 65 years, with a history of lung cancer, the initial diameter of SSN ≥ 8 mm, and the presence of air bronchus signs. Continued follow-up of SSNs with different diameters stable for five years has found that some SSNs still grow. Even SSNs that have been stable for five years and have a diameter of <6 mm in low-risk populations still require long-term follow-up (36, 37). Related studies on persistent and steady SSNs are summarized in **Table 2**.

## 6 Follow-up strategy

Appropriate follow-up and management of SSN can provide evidence for early detection of lung cancer, thereby improving patient outcomes. Most current guidelines formulate follow-up strategies based primarily on SSN diameter, solid composition, and growth rate (**Table 3**).

**Table 3:** Differences in follow-up strategies of SSN in guidelines.

Guideline	pGGN diameter				PSN diameter			
	<6mm	≥6mm	<20mm^a^/30mm^b^	≥20mm^a^/30mm^b^	<6mm	≥6mm	<8mm	≥8mm
**2022 NCCN NSCLC Guidelines**	No follow-up needed	CT at 6-12 month to confirm persistence, then CT every 2 years until 5 years			No follow-up needed	CT at 3-6 month to confirm persistence; If unchanged and solid component < 6 mm, annual CT should be performed for 5 years; If the solid component ≥ 6mm, then PET-CT or surgical resection.		
**2017 Fleischner Society Guidelines**	No follow-up needed	CT at 6-12 month to confirm persistence, then CT every 2 years until 5 years			No follow-up needed	**For solid component <5mm** CT at 3-6 month to confirm persistence, then annual CT until 5 years **For solid component ≥5mm** CT at 3-6 month to confirm persistence, PET-CT/biopsy/resection are recommended for the persistence of the nodule.		
**2013 ACCP Guidelines**	No follow-up needed	Annual CT for at least 3 years; If diameter>10mm, early follow-up at 3 months, followed by nonsurgical biopsy and/or surgical resection for nodules that persist.					CT at 3, 12, 24 month, and then annual CT for at 1-3 years	CT at 3 month to confirm persistence; For persistent, biopsy/surgical resection; For nodule >15 mm at first CT scan, biopsy/PET-CT/surgical resection.
**2016 Clinical** **practice** **consensus** **guidelines** **for Asia**	Discuss the role of continued surveillance with patient	Annual CT for at least 3 years					CT at 3, 6, 12 month, and then annual CT surveillance	CT at 3 month, and consider antimicrobial therapy (nonsurgical or surgical biopsy consider PET scanning for staging before biopsy)
**2022 NCCN LCS Guidelines**			Annual screening LDCT until the patient is no longer a candidate for definitive treatment; For stable, annual LDCT; For growth(>1.5mm), review at 6 months	LDCT in 6 month; For stable, annual LDCT; For growth(>1.5mm), review at 6 months or consider biopsy or surgical excision	Annual screening LDCT until the patient is no longer a candidate for definitive treatment;	**For solid component <6mm** LDCT at 6 month to confirm unchange, then annual LDCT		
						**For solid component ≥6 to <8mm** LDCT in 3 month or consider PET-CT; if unchanged, LDCT at 6 moth, then annual CT Low suspicion of lung cancer, LDCT in 3 month; Low suspicion of lung cancer, biopsy or resection		
						**For solid component >8mm** Chest CT+ contrast and/or PET-CT; Low suspicion of lung cancer, LDCT in 3 month; Low suspicion of lung cancer, biopsy or resection		
					**Newly developed nodules** LDCT in 6 month	**Newly developed nodules** If solid component <4mm, LDCT in 3 month; If solid component ≥4mm, Chest CT+ contrast and/or PET-CT; Low suspicion of lung cancer, LDCT in 3 month; High suspicion of lung cancer, biopsy or resection.		
**2019 Lung-RADS^b^**			Continue annual screening with LDCT in 12 months.	≥ 30 mm and unchanged or slowly growing: Continue annual screening with LDCT in 12 months. ≥ 30 mm on baseline CT or new: 6 month LDCT; unchanged for ≥ 3 months, then annual screening with LDCT in 12 months.	Continue annual screening with LDCT in 12 months.	With solid component < 6 mm OR new < 6 mm total diameter: 6 month LDCT; unchanged for ≥ 3 months, then annual screening with LDCT in 12 months. With solid component ≥ 6 mm to < 8 mm OR with a new or growing < 4 mm solid component: 3 month LDCT; PET/CT may be used when there is a ≥ 8 mm solid component; Unchanged for ≥ 3 months, then annual screening with LDCT in 12 months.		a solid component ≥ 8 mm OR a new or growing ≥ 4 mm solid component: Chest CT with or without contrast, PET/CT and/or tissue sampling depending on the probability of malignancy and comorbidities; unchanged for ≥ 3 months, then annual screening with LDCT in 12 months. For new large nodules that develop on an annual repeat screening CT, a 1 month LDCT may be recommended to address potentially infectious or inflammatory conditions.
								

pGGN, pure ground-glass nodule; mGGN, mixed ground-glass nodule; NCCN, National Comprehensive Cancer Network; NSCLC, Non-Small Cell Lung Cancer; LCS, Lung Cancer Screening; ACCP, American College of Chest Physicians; CT, computed tomography; LDCT, low-dose computed tomography; PET-CT, Positron Emission Tomography-Computed Tomography; Lung-RADS, Lung imaging reporting and data system; ^a^indicates that 2022 NCCN LCS Guidelines managed pGGN with a limit of 20mm; ^b^indicates that 2019 Lung-RADS managed pGGN with a limit of 30mm.

### 6.1 Solitary pGGN

For solitary pGGN <6 mm, National Comprehensive Cancer Network (NCCN) Guidelines for Non-Small Cell Lung Cancer, 2017 Fleischner Association Guidelines (21), and American College of Chest Physicians (ACCP) Guidelines (64) all recommend that no follow-up is required. The Asian Clinical Practice Consensus (65) recommended an annual review. The NCCN lung cancer screening guidelines take 20mm as the boundary. It is recommended that the baseline presence of <20mm and the new pGGN should be reviewed every year, and the enlarged (>1.5mm) should be reviewed every six months.

For solitary pGGN ≥6 mm, the NCCN and Fleischner guidelines recommend CT at 6-12 months to confirm no enlargement or presence of a physical component, and then every two years for five years. The difference between the ACCP and NCCN guidelines is that the former requires a continuous 3-year review, and an annual CT is still required if the nodule does not change. In the screening guidelines, the NCCN states that ≥20mm baseline presence and new pGGN should be reviewed after six months, and stable should be reviewed annually.

### 6.2 Solitary PSN

The NCCN and Fleischner Guidelines recommend no routine follow-up for PSNs <6 mm in diameter. The ACCP guidelines and the Asian Clinical Consensus recommend that PSNs with a diameter of less than 8 mm should be reviewed at 3, 12, and 24 months, followed by an annual review at 1-3 years and 3, 6, and 12 months, followed by a yearly review. NCCN lung cancer screening recommends an annual review of PSN with <6mm baseline until the patient is no longer a potential target for lung cancer treatment, and new nodules need to be reviewed after six months.

For ≥6 mm PSN, the NCCN Guidelines recommend CT at 3-6 months to confirm no growth or changes in the solid component, followed by an annual five-year review. The Fleischner guidelines are based on whether the substantial part is less than 5 mm. The former requires an initial assessment of 3-6 months, followed by an annual review for a minimum of 5 years; the latter requires 3-6 months of review, and PET/CT, tissue biopsy, or surgical resection. The ACCP guidelines and the Asian Clinical Consensus recommend reexamination every three months. If persistent, it is advised to confirm the diagnosis. At the same time, the ACCP guidelines directly perform PET-CT/biopsy/surgery for >15mm PSN. In the screening guidelines, the NCCN guidelines recommend that the solid component diameter <6 mm can be re-examined after six months; after ≥ 6 mm, the re-examination can be performed after three months. Among them, the NCCN guidelines pointed out that the diameter of the solid component of the newly issued PSN is less than 4 mm, and it can be re-examined after three months; if it is greater than 4 mm, the diagnosis needs to be confirmed.

### 6.3 Multiple SSN

There are still few guidelines for imaging follow-up strategies for multiple SSNs. Among them, the NCCN non-small cell lung cancer guidelines and the Fleischner Association guidelines recommend: that if the diameter of SSN is less than 6mm, CT examination should be performed in 3-6 months and stable in the second year. And the 4th year review, if the diameter of the SSN is ≥6mm, a CT scan should be reviewed in 3-6 months, and management should be based on the most suspicious nodules. If the nodules still exist, consider multiple primary adenocarcinomas.

BTS (British Society Thoracic Society) guidelines (22) do not categorize nodule types and focus on nodule diameter and risk assessment models for nodule management.

### 6.4 Lung imaging reporting and data system

The American College of Radiology (ACR) proposed the Lung imaging reporting and data system (Lung-RADs) version 1.1 based on linear measurement in 2019, aiming to standardize and improve the accuracy of GGN screening and interpretation (66). This allows for more precise management of pulmonary nodules. Kim et al. found a significant difference in Lung-RADs scores between pre-existing PSNs and newly emerged PSNs, and that pre-existing PSNs had a higher lung cancer diagnosis rate compared with new-onset PSNs (13). Hammer et al. used lung-Rads to evaluate SSNS detected by NLST Lung cancer CT screening to verify their efficacy and found that GGNs smaller than 10mm had a lower malignity rate than GGNs smaller than 10-19mm. The risk of malignancy in both categories 2 and 3 of Lung-RADS is higher than that recommended by expert opinion-based Lung-RADs (67). However, the implications for the management of SSN remain uncertain, as these nodules often exhibit inactivity when malignant in nature and require a long-term follow-up plan. Hui-Ting Hsu et al. found that category 2 and 3 of Lung-RADs were modified to include subcategories 2A/2B/2C and 3A/3B/3C, respectively, and found that the modified Lung-RADS could significantly improve the sensitivity while maintaining specificity for detection of adenocarcinoma lineage lesions in Asian populations (68).

The Lung-RADS classification of ACR is basically the same as the NCCN guidelines. The biggest difference from the Asian population is that ACR believes that all ground-glass nodules <30mm are low-risk, at most grade 2, with less than a malignancy risk. 1% and neither is active cancer. The main rationale is that carcinoma *in situ* and minimally invasive adenocarcinoma are not life-threatening. Therefore, in the grading of ACR, early-stage lung cancer manifested by ground glass nodules is not included in the category of malignant probability due to its indolent biological manifestations. Of course, the grading system also has imperfections. The grading system is mainly used by family community doctors in the United States. Moreover, pulmonary nodules are ever-changing, and cultural differences between China and the United States will also lead to very different treatment strategies for pulmonary nodules.

### 6.5 Challenges of guidelines

In addition to guidelines from Western countries, China, Japan (69), and South Korea (70) have national guidelines. In contrast, healthcare practitioners can refer to individual, institutional standard, or Western guidelines in other countries. However, the fact is that the above guidelines do not appear to be commonly used in practice, and most clinicians tend to manage those detected nodules based on their own experience in interpreting CT images and the personality of the individual patient, even in countries with guidelines. Especially in Asian populations, there is an increase in the prevalence of SSN due to high LDCT use, and the risk of lung cancer and potential overdiagnosis associated with non-smoking has been increasing in recent years. Therefore, management decisions need to be balanced between early lung cancer detection and overdiagnosis.

The reasons for this analysis can be classified into the following three points: (1) Practice guidelines may not apply to patients whose risk of malignancy differs from that of the general population. For example, patients with malignancy or a recent history of malignancy; patients with organ transplantation or other immunocompromised states; patients aged <35 years. For these individuals, referral to a pulmonologist or a multidisciplinary pulmonary nodule specialist clinic may be required. (2) The definitions and trends of high-risk groups in each lung cancer screening guideline differ significantly among different ethnic groups in the East and the West (71), as mentioned the screening criteria for the NLST are not applicable to the Chinese population other than the United States (72). They also differ in different group cohorts of the same ethnicity (73). (3) Overdiagnosis and treatment due to concerns about the risk of death (74). Some patients prefer to undergo resection of small SSN with some potential for malignancy due to anxiety, costs, and radiation exposure involved in long-term monitoring of SSN, rather than following a wait-and-see policy, increasing the likelihood of overdiagnosis. In fact, in non-smoking Asian populations, SSN has a relatively high rate of indolent behavior (75). (4) LDCT screened small pulmonary SSNs, which were missed and misdiagnosed due to the doctor’s management experience, resulting in the tumor progressing to advanced lung cancer. (5) Currently, many specialists from different backgrounds, including pulmonology, radiology, thoracic surgery, and family medicine, use the concept of shared decision-making (SDM) with the patient to manage screening nodules (76, 77), including ongoing randomized trials (78). In practice, different specialists and their associated medical societies may not adopt guidelines from other organizations, or guidelines may not exist in their communities.

### 6.6 Future directions of guidelines

**Figure 4:**
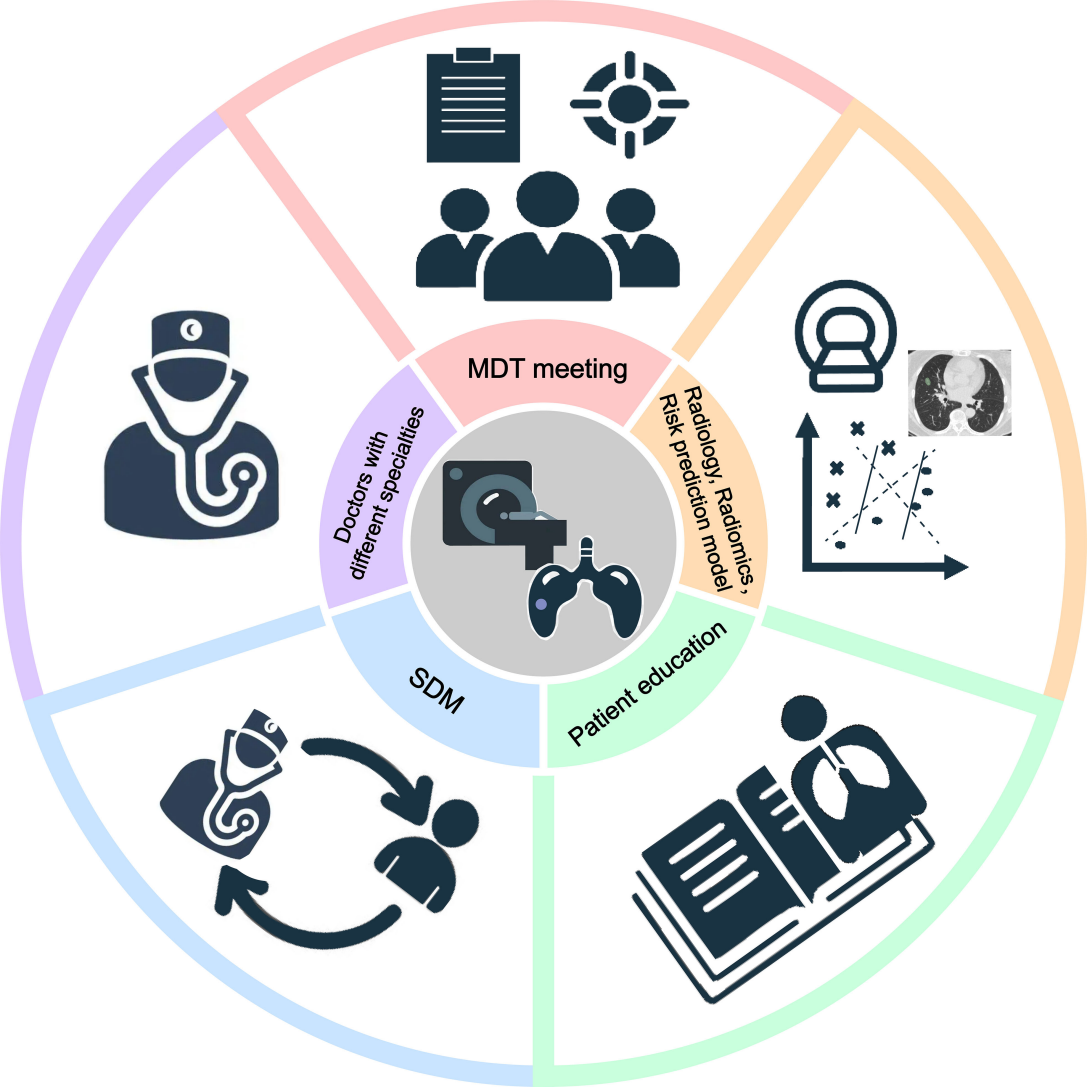
The vital factors in the individualized management of persistent SSNs. Five key factors are required for the individualized management of persistent SSNs, including doctors with different specialties, MDT(multidisciplinary team) meeting, radiology, radiomics, risk prediction model, SDM (share decision-making) and patient education.

Underdiagnosis, overdiagnosis and excessive management cannot be avoided, so how to solve the above-mentioned problems: (1) The large-scale lung cancer screening mentioned in the guideline can be transformed into a targeted screening of high-risk groups. For example, in the guidelines for the Asian population, non-smoking people are included in lung cancer screening, and family history of lung cancer and female gender are used as screening criteria for non-smokers to establish a specific screening plan (72). (2) In addition to classification management by nodule diameter and solid component size, the original and modified Lung-RADS were used for group management according to different ethnic groups (68). A multi-dimensional nodule risk stratification model and whole-process management system were established to achieve accurate management and reduce overdiagnosis. (3) Longitudinal follow-up of suspected precancerous lesions in screening programs is an effective strategy for active surveillance to prevent overdiagnosis. (4) Establish a large-sample lung cancer intelligent database, use artificial intelligence to read images to solve the bottleneck of missed diagnosis of early lung cancer, and improve the early diagnosis rate of lung cancer. Ideally, each patient with SSN should be discussed at an MDT meeting to determine optimal management and follow-up strategies for SSN (**Figure 4**).

## 7 Molecular pathological features of SSN growth

Early-stage LUAD with SSN as imaging manifestation is a relatively indolent tumor with a reasonable survival rate (79–81). The clinical and imaging features related to the natural history of SSN have been extensively studied, but the molecular pathological features of its growth are still worthy of further exploration. Kobayashi et al. (82) included 104 cases of SSN with CTR ≥ 50% and pathologically confirmed as early lung adenocarcinoma and analyzed their preoperative imaging data and genetic testing results. Compared with SSN, EGFR mutation-positive SSN pathological types were associated with MIA/IA. EGFR/KRAS/ALK/HER2 mutation-negative SSN remained stable, and the pathological type tended to be AAH/AIS. Studies by Li et al. (83) showed that with the increase in the solid component of SSN, the lesions were significantly enriched for EGFR, TP53, RBM10, and ARID1B mutations, suggesting that these gene mutations play an essential role in the malignant progression of SSN to lung adenocarcinoma. The natural growth characteristics of SSN reflect the heterogeneity within the tumor, and studying related factors of SSN growth from the perspective of genomics may become a feasible way to predict the growth of GGN in the future. It is worth noting that the diameter of SSNs discovered early is usually tiny, and obtaining genetic test results in clinical diagnosis and treatment isn’t easy. Its clinical application still relies on the breakthrough of gene mutation prediction through radiomics and liquid biopsy research.

## 8 The application of new technology in SSN growth assessment

### 8.1 Radiomic

Radiomics aims to extract large amounts of quantifiable information from images in an automated (or semi-automated) way, correlate radiographic images with intrinsic heterogeneity, genetic characteristics, or other phenotypes, and develop models to predict in a non-invasive manner lesion phenotypes to improve disease outcomes (84). Up to now, there have been many radiomics studies to establish predictive models to explore the judgment of benign and malignant nodules, the interpretation of the degree of invasion and histopathological subtypes, and the prognosis of lung cancer patients, to improve the diagnostic accuracy of early lung cancer (85). However, it is difficult to make great progress in how to use radiomics to dynamically track nodules and predict the interphase growth of SSN due to the limitation of the number of cases and the follow-up time. Li et al. developed a radiomics nomogram to predict 2-year pulmonary nodule growth for nodules that CT could not identify. Of 215 pulmonary nodules (182 SSN) included in the study, 109 grew within two years, and 106 remained stable for more than two years. 1316 features were extracted from ultra-high-resolution CT target scan images, 11 features were selected to construct radiomics features, and they were combined with clinical features to establish a radiomics nomogram for predicting the 2-year growth of nodules. Radiomics nomogram had a higher AUC (0.911 95%CI: 0.867) than radiomics (0.892 95%CI: 0.843–0.940) and clinical (0.812 95%CI: 0.747–0.877) (49). Tan et al. (86) included 402 patients with pathologically confirmed early-stage lung adenocarcinoma with two or more thin-slice CT follow-up images, a total of 407 nodules, of which 325 were SSN. The imaging feature-radiomics model was established to predict nodule growth velocity, and the results showed that the combined imaging feature-radiomics model (AUC 0.780) outperformed the imaging feature model (0.727) and the radiomics model (0.710). Chen et al. (34) included 85 patients with 110 SSNs, including diameter and five specific radiomic features, to establish clinical and radiomic models. The results showed that the radiomic characteristic model (RAD-Score) and diameter were considered predictors of GGN growth, and the AUC of the clinical-radiomic combined model reached 0.801. Therefore, a relevant model based on clinical features and radiomics were established to predict nodule growth in a non-invasive manner, thereby effectively improving the management of PSN.

### 8.2 Artificial intelligence

Deep learning transforms first-level representations into higher, more abstract terms by combining simple but non-linear modules. It is a specific type of machine learning and part of AI (87, 88). Compared with radiomics, deep learning reduces physician labeling workload and automatically extracts high-order features. Qi et al. research based on the AI-assisted system Dr. Wise found that compared with the two-dimensional diameter parameter, the three-dimensional volume parameter can reflect the growth of SSN with higher sensitivity and accuracy (28, 35). Tao et al. found that building a visual prediction system based on deep learning can also accurately predict future images of SSN (89). However, deep learning has certain limitations. For example, it requires a high amount of data, and the process of learning features inside the model is like a “black box”. At present, there are still few related studies on the prediction of SSN growth based on artificial intelligence. In the future, the deep learning SSN growth risk assessment model deserves further development and needs to be verified in different populations in clinical applications.

## 9 Future directions to explore

At present, the concepts of radiomics and machine learning are on the ascendant, and many technology companies have invested in the research of AI-assisted imaging diagnosis. A large imaging dataset supports the application of AI to identify lung nodules most likely to grow, pathologically suggestive of malignancy. Improved management of indeterminate pulmonary nodule (IPN) by training a lung cancer-predicting convolutional neural network model using more than 15,000 images of IPN from the NLST (National Lung Screening Trial), which externally validated to demonstrate superior advantages over currently available risk prediction models (90). How to effectively complete the cross-section of SSN, that is, how to judge the benign and malignant and predict the pathological type, and how to predict the interval growth of high-risk population through radiomics and AI, so as to achieve dynamic and efficient management during follow-up is a clinically important problem, which also needs further research.

Biomarkers are objectively observable indicators to evaluate normal physiological and pathological processes (91). Using reliable biomarkers can better identify high-risk populations and better aid in identifying SSN properties, thereby improving the management of pulmonary nodules (92). “Liquid biopsy” technology has gradually attracted attention as a non-invasive examination method. Exosomes, circulating tumor cells (CTC), circulating free DNA (cfDNA), circulating tumor DNA (ctDNA), and DNA methylation have been used in clinical evaluation (93–98). How to predict whether SSN will progress and the rate of progression of lung cancer with SSN as the primary manifestation? Currently, studies have tried to predict by single or multi-omics methods such as essential clinical characteristics of patients and radiomics. Still, there is no reliable model to answer this question. The molecular events in the progression of SSN also need to be further explored.

## 10 Conclusion

Persistent SSNs grow exponentially and behave indolently compared to solid nodules, so long-term active monitoring is a safe strategy to reduce overtreatment. Initial diameter, previous history of malignancy, and some imaging features are important risk factors for SSN growth. For persistent and stable SSN, follow-up should be at least five years while focusing on the population with high-risk factors. The ethnic groups on which major guidelines formulate the SSN guidelines differ, and the follow-up strategies differ. An MDT team needs to develop follow-up and management strategies for nodules. The molecular mechanism of SSN growth deserves further exploration. Predicting and quantitatively evaluating the growth of GGN based on clinical and imaging feature data can provide a reference for the formulation of clinical diagnosis and treatment strategies for GGN patients and has significant clinical application value.

## Author contributions

Conceptualization, data curation, and original draft preparation were done by ZZ, LZ, FY, and XL. Critical revision of the manuscript for important intellectual content was done by XL. All authors contributed to the article and approved the submitted version.
